# Discontinuous Mixed Covolume Methods for Parabolic Problems

**DOI:** 10.1155/2014/867863

**Published:** 2014-03-30

**Authors:** Ailing Zhu, Ziwen Jiang

**Affiliations:** School of Mathematical Sciences, Shandong Normal University, Jinan 250014, China

## Abstract

We present the semidiscrete and the backward Euler fully discrete discontinuous mixed covolume schemes for parabolic problems on triangular meshes. We give the error analysis of the discontinuous mixed covolume schemes and obtain optimal order error estimates in discontinuous *H*(div) and first-order error estimate in *L* ^2^.

## 1. Introduction

The study of discontinuous Galerkin methods has been a very active research area since its introduction in [1] in 1973. The discontinuous Galerkin method does not require continuity of the approximation functions across the interelement boundary but instead enforces the connection between elements by adding a penalty term. Because of the use of discontinuous functions, discontinuous Galerkin methods have the advantages of a high order of accuracy, high parallelizability, localizability, and easy handling of complicated geometries. Discontinuous Galerkin methods have been used to solve hyperbolic and elliptic equations by many researchers. For example, see [2–10]. In [11], the unified analysis of discontinuous Galerkin methods for elliptic problems was presented. In [12, 13], Ye developed a new discontinuous finite volume method for elliptic and Stokes problems, respectively. The discontinuous finite volume method was used for parabolic equations by Bi and Geng in [14]. In [15], Yang and Jiang extended a new discontinuous mixed covolume method for elliptic problems. In this paper, we consider the semidiscrete and the backward Euler fully discrete discontinuous mixed covolume methods for the second-order parabolic problems and derive the optimal order error estimates in the discontinuous *H*(div⁡) and first-order *L* ^2^-error estimates in a mesh-dependent norm.

The rest of this paper is organized as follows. In Section 2, we introduce some notations and describe the discontinuous mixed covolume schemes for the second-order parabolic problems and give some lemmas which will be used in the convergence analysis. In Section 3, we prove the existence and uniqueness for the semidiscrete and the backward Euler fully discrete discontinuous mixed covolume approximation. A discontinuous mixed covolume elliptic projection is defined in Section 4. Error estimations in both discontinuous *H*(div⁡) and *L* ^2^ norms of semidiscrete method and fully discrete method are proved in Sections 5 and 6.

Throughout this paper, letter *C* denotes a generic positive constant independent of the mesh parameter and may stand for different values at its different appearances.

## 2. Discontinuous Mixed Covolume Formulation

In this paper, we consider the following parabolic problems:

(1) pt(x,t)−∇·{D(x,t)∇p(x,t)}=f(x,t),(x,t)∈Ω×(0,T],p(x,0)=p0(x), x∈Ω,p(x,t)=0, (x,t)∈∂Ω×(0,T],

where *Ω* ∈ **R** ^2^ is a bounded convex polygonal domain with the boundary ∂*Ω*,  **x** = (*x*, *y*),  *p* is an unknown function, and *D* is a symmetric, bounded matrix function which satisfies the following condition: there exist two positive constants *α*_1_, *α*_2_ such that

(2) $$\begin{array}{c} \alpha_{1}\xi^{⊤}\xi \leq \xi^{⊤}D\xi \leq \alpha_{2}\xi^{⊤}\xi ,\;\forall \xi =\left(\xi_{1},\xi_{2}\right)\in R^{2}; \end{array}$$

*f* is a given function in *L* ^2^(*Ω*). Furthermore, we assume that the matrix *M* = *D* ^−1^ is locally Lipschitz.

Here and in what follows, we will not write the independent **x**, *t* for any functions unless it is necessary.

Let **u** = −*D*∇*p*, and rewrite (1) as the system of first-order partial differential equations:

(3) $$\begin{array}{c} Mu+\nabla p=0,\;\left(x,t\right)\in \Omega \times \left(0,T\right],\\ p_{t}+\nabla \cdot u=f,\;\left(x,t\right)\in \Omega \times \left(0,T\right],\\ p\left(x,0\right)=p_{0}\left(x\right),\;x\in \Omega ,\\ p\left(x,t\right)=0,\;\left(x,t\right)\in \partial \Omega \times \left(0,T\right]. \end{array}$$

We will use the standard definitions for the Sobolev spaces *H* ^*s*^(*K*) and their associated inner products (·, ·)_*s*,*K*_, norms ||·||_*s*,*K*_, and seminorms |·|_*s*,*K*_. The space *H* ^0^(*K*) coincides with *L* ^*s*^(*K*), in which the norm and the inner product are denoted by ||·||_*K*_ and (·, ·)_*K*_, respectively.

Let *𝒯*_*h*_ = {*K*} be a triangulation of the domain *Ω*. As usual, we assume the triangles *K* to be shape-regular. For a given triangulation *𝒯*_*h*_, we construct a dual mesh *𝒯*_h_* based upon the primal partition *𝒯*_*h*_. Each triangle in *𝒯*_*h*_ can be divided into three subtriangles by connecting the barycenter *Q* of the triangle to their corner nodes *A*_*i*_  (*i* = 1,2, 3). Then, we define the dual partition *𝒯*_h_* to be the union of the triangles. Let *P*_*k*_(*T*) consist of all the polynomials functions of degree less than or equal to *k* defined on *T*. We define the finite-dimensional trial function space for velocity on *𝒯*_*h*_ by

(4) $$\begin{array}{c} V_{h}:=\left\{v\in L^{2}{\left(\Omega \right)}^{2}:{v|}_{K}\in P_{1}{\left(K\right)}^{2},\forall K\in {𝒯}_{h}\right\}. \end{array}$$

Define the finite-dimensional test function space *W*_*h*_ for velocity associated with the dual partition *𝒯*_h_* as

(5) $$\begin{array}{c} W_{h}:=\left\{w\in L^{2}{\left(\Omega \right)}^{2}:{w|}_{T}\in P_{0}{\left(T\right)}^{2},\forall T\in {𝒯}_{h}^{*}\right\}. \end{array}$$

Let *H*_*h*_ be the finite-dimensional space for pressure:

(6) $$\begin{array}{c} H_{h}:=\left\{q\in L^{2}\left(\Omega \right):{q|}_{K}\in P_{0}\left(K\right),\forall K\in {𝒯}_{h}\right\}. \end{array}$$

Let Γ denote the union of the boundary of the triangles *K* of *𝒯*_*h*_ and Γ^0^ : Γ∖∂*Ω*. The traces of functions in *V*_*h*_ and *H*_*h*_ are double valued on Γ^0^. Let *e* be an interior edge shared by two triangles *K*_1_ and *K*_2_ in *T*_*h*_. Define the normal vectors **n**_1_ and **n**_2_ on *e* pointing exterior to *K*_1_ and *K*_2_, respectively. Next, we introduce some traces operators that we will use in our numerical formulation. We define the average {·} and jump [·] on *e* for scalar *q* and vector **v**, respectively,

(7) $$\begin{array}{c} \left\{q\right\}=\frac{1}{2}\left({q|}_{\partial K_{1}}+{q|}_{\partial K_{2}}\right),\;\;\left[q\right]={q|}_{\partial K_{1}}n_{1}+{q|}_{\partial K_{2}}n_{2},\\ \left\{v\right\}=\frac{1}{2}\left({v|}_{\partial K_{1}}+{v|}_{\partial K_{2}}\right),\;\;\left[v\right]={v|}_{\partial K_{1}}n_{1}+{v|}_{\partial K_{2}}n_{2}. \end{array}$$

If *e* is an edge on the boundary of *Ω*, we set

(8) $$\begin{array}{c} \left\{q\right\}=q,\;\;\left[v\right]=v\cdot n, \end{array}$$

where **n** is the outward unit normal. We do not require either of the quantities [*q*] or {**v**} on boundary edges, and we leave them undefined.

Multiplying the first and second equations in system (3) by **w** ∈ *W*_*h*_ and *q* ∈ *H*_*h*_, respectively, and using the integration by parts formula in the equation, we have

(9) $$\begin{array}{c} \underset{T\in {𝒯}_{h}^{*}}{\sum }\int_{T}Mu\cdot w\;dx+\underset{T\in {𝒯}_{h}^{*}}{\sum }\int_{\partial T}pw\cdot n\;ds=0,\;\forall w\in W_{h},\\ \underset{K\in {𝒯}_{h}}{\sum }\int_{K}p_{t}q\;dx+\underset{K\in {𝒯}_{h}}{\sum }\int_{K}\nabla \cdot uq\;dx=\left(f,q\right),\;\forall q\in H_{h}, \end{array}$$

where **n** is the outward normal vector on ∂*T*. Let *T*_*j*_ ∈ *𝒯*_h_*  (*j* = 1,2, 3) be the triangles in *K* ∈ *𝒯*_*h*_. Then we have

(10) ∑T∈𝒯h∗∫∂Tpw·n ds=∑K∈𝒯h ∑j=13∫Aj+1QAjpw·n ds+∑K∈𝒯h∫∂Kpw·n ds, ∀w∈Wh,

where *A*_4_ = *A*_1_. A straightforward computation gives

(11) ∑K∈𝒯h∫∂Kpw·n ds=∑e∈Γ0∫e[p]·{w}ds+∑e∈Γ∫e{p}·[w]ds, ∀w∈Wh.

Let ∫_Γ_ *p* *ds* = ∑_*e*∈Γ_∫_*e*_ *p* *ds*. Using the above formula and the fact that [*p*] = 0 for *p* ∈ *H* ^1^(*Ω*) on Γ^0^, (10) becomes

(12) ∑T∈𝒯h∗∫∂Tpw·n ds=∑K∈𝒯h ∑j=13∫Aj+1QAjpw·n ds+∑e∈Γ∫e{p}[w]ds, ∀w∈Wh.

Then, system (9) can be rewritten as follows:

(13) ∑T∈𝒯h∗∫TMu·w dx+∑K∈𝒯h ∑j=13∫Aj+1QAjpw·n ds  +∑e∈Γ∫e{p}[w]ds=0, ∀w∈Wh,∑K∈𝒯h∫Kptq dx+∑K∈𝒯h∫K∇·uq dx=(f,q), ∀q∈Hh.

Let *V*(*h*) = *V*_*h*_ + *H* ^2^(*Ω*)^2^. Define a mapping *γ* : *V*(*h*) → *W*_*h*_ as

(14) $$\begin{array}{c} {\gamma v|}_{T}=\frac{1}{h_{e}}\int_{e}{v|}_{T}ds,\;T\in {𝒯}_{h}^{*}, \end{array}$$

where *h*_*e*_ is the length of the edge *e*. For **v** = (*v*_1_, *v*_2_) ∈ *V*(*h*), *γv*_*i*_  (*i* = 1,2) is defined as

(15) $$\begin{array}{c} {\gamma v_{i}|}_{T}=\frac{1}{h_{e}}\int_{e}{v_{i}|}_{T}ds,\;T\in {𝒯}_{h}^{*},\;\;\left(i=1,2\right). \end{array}$$

Then the system (13) is equivalent to

(16) ∑T∈𝒯h∗∫TMu·γw dx+∑K∈𝒯h ‍∑j=13∫Aj+1QAjpγw·n ds  +∑e∈Γ∫e{p}[γw]ds=0, ∀w∈V(h),∑K∈𝒯h∫Kptq dx+∑K∈𝒯h∫K∇·uq dx=(f,q), ∀q∈Hh.

Let

(17) $$\begin{array}{c} a_{0}\left(v,w\right):=\underset{T\in {𝒯}_{h}^{*}}{\sum }\int_{T}Mv\cdot \gamma w\;dx,\\ b\left(w,q\right):=\underset{K\in {𝒯}_{h}}{\sum }\;\overset{3}{\underset{j=1}{\sum }}\int_{A_{j+1}QA_{j}}q\gamma w\cdot n\;ds+\underset{e\in \Gamma }{\sum }\int_{e}\left\{q\right\}\left[\gamma w\right]ds,\\ c_{0}\left(v,q\right):=\underset{K\in {𝒯}_{h}}{\sum }\int_{K}\nabla \cdot vq\;dx. \end{array}$$

Using the above bilinear forms, it is clear that system (16) can be rewritten as follows:

(18) $$\begin{array}{c} a_{0}\left(u,w\right)+b\left(w,p\right)=0,\;\forall w\in V\left(h\right),\\ \left(p_{t},q\right)+c_{0}\left(u,q\right)=\left(f,q\right),\;\forall q\in H_{h}. \end{array}$$

In order to define our numerical schemes, we introduce the bilinear forms as follows:

(19) $$\begin{array}{c} a\left(v,w\right):=a_{0}\left(v,w\right)+\alpha \underset{e\in \Gamma }{\sum }\frac{1}{h_{e}}\int_{e}M\left[v\right]\left[w\right]ds,\\ c\left(v,q\right):=c_{0}\left(v,q\right)-\int_{\Gamma }\left\{q\right\}\left[\gamma v\right]ds, \end{array}$$

where *α* > 0 is a parameter to be determined later. For the exact solution (**u**, *p*) of system (3), we have

(20) $$\begin{array}{c} a_{0}\left(u,v\right)=a\left(u,v\right),\;\forall v\in V_{h},\\ c_{0}\left(u,q\right)=c\left(u,q\right),\;\forall q\in H_{h}. \end{array}$$

Therefore, it follows from (18) that

(21) $$\begin{array}{c} a\left(u,w\right)+b\left(w,p\right)=0,\;\forall w\in V_{h},\\ \left(p_{t},q\right)+c\left(u,q\right)=\left(f,q\right),\;\forall q\in H_{h}. \end{array}$$

The discontinuous mixed covolume scheme for (3) reads as follows. Seek (**u**_*h*_, *p*_*h*_) ∈ *V*_*h*_ × *H*_*h*_ such that

(22) $$\begin{array}{c} a\left(u_{h},w\right)+b\left(w,p_{h}\right)=0,\;\forall w\in V_{h},\\ \left(p_{ht},q\right)+c\left(u_{h},q\right)=\left(f,q\right),\;\forall q\in H_{h}, \end{array}$$

where $p_{h}(0)={\tilde{p}}_{h}(0)$, $u_{h}(0)={\tilde{u}}_{h}(0)$, ${\tilde{p}}_{h}(0)$, ${\tilde{u}}_{h}(0)$ will be given in Section 4.

Let *N* > 0 be a positive integer, let 0 = *t* ^0^ < *t* ^1^ < ⋯<*t* ^*j*^ < ⋯<*t* ^*N*^ = *T* be a subdivision of time. *t* ^*j*^ = *j*Δ*t*  (0 ≤ *j* ≤ *N*), Δ*t* = *T*/*N*. We use the backward Euler difference quotient

(23) $$\begin{array}{c} \partial_{t}p_{h}^{j}=\frac{p_{h}^{j}-p_{h}^{j-1}}{\Delta t},\;\left(j=1,2,\ldots ,N\right) \end{array}$$

to approximate the differential quotient (∂*p*_*h*_^*j*^/∂*t*)  (*j* = 1,2,…, *N*), in the semidiscrete scheme; then we obtain the backward Euler fully discrete discontinuous mixed covolume scheme for the problem (1): find (**u**_*h*_^*j*^, *p*_*h*_^*j*^) ∈ *V*_*h*_ × *H*_*h*_,  (*j* = 1,2,…, *N*), such that

(24) $$\begin{array}{c} a\left(u_{h}^{j},w\right)+b\left(w,p_{h}^{j}\right)=0,\;\forall w\in V_{h},\\ \left(\partial_{t}p_{h}^{j},q\right)+c\left(u_{h}^{j},q\right)=\left(f,q\right),\\ \forall q\in H_{h},\;\left(j=1,2,\ldots ,N\right), \end{array}$$

where $p_{h}^{0}={\tilde{p}}_{h}(0)$, $u_{h}^{0}={\tilde{u}}_{h}(0),{\tilde{p}}_{h}(0),{\tilde{u}}_{h}(0)$ will be given in Section 4.

We define the following norms for **v** ∈ *V*(*h*):

(25) $$\begin{array}{c} {\left|\left|\left|v\right|\right|\right|}_{\mathrm{div}}^{2}={||v||}^{2}+{||\nabla_{h}\cdot v||}^{2}+\underset{e\in \Gamma }{\sum }\frac{1}{h_{e}}\int_{e}{\left[v\right]}^{2}ds,\\ {\left|\left|\left|v\right|\right|\right|}_{1}^{2}={||v||}^{2}+{\left|v\right|}_{1,h}^{2}+\underset{e\in \Gamma }{\sum }\frac{1}{h_{e}}\int_{e}{\left[v\right]}^{2}ds,\\ {\left|\left|\left|v\right|\right|\right|}_{}^{2}={\left|\left|\left|v\right|\right|\right|}_{1}^{2}+\underset{K\in {𝒯}_{h}}{\sum }h_{K}^{2}{\left|v\right|}_{2,K}^{2}, \end{array}$$

where ∇_*h*_ · **v** is the function whose restriction to each element *K* ∈ *𝒯*_*h*_ is equal to ∇·**v**, and |**v**|_1,*h*_^2^ = ∑_*K*∈*𝒯*_*h*__|**v**|_1,*K*_^2^.

We will introduce some useful lemmas; for more details, see [6].

**Lemma 1**

For **v**, **w** ∈ *V*(*h*), one has

(26) $$\begin{array}{c} a\left(v,w\right)\leq C{\left|\left|\left|v\right|\right|\right|}_{\mathrm{div}}^{}{\left|\left|\left|w\right|\right|\right|}_{\mathrm{div}}^{}. \end{array}$$

**Lemma 2**

For (**v**, *q*) ∈ *V*(*h*) × *L* ^2^(*Ω*), one has

(27) $$\begin{array}{c} b\left(v,q\right)=-c\left(v,q\right). \end{array}$$

**Lemma 3**

For (**v**, *q*) ∈ *V*(*h*) × *L* ^2^(*Ω*), one has

(28) $$\begin{array}{c} b\left(v,q\right)\leq C\left|\left|\left|v\right|\right|\right|\left(||q||+{\left(\overset{}{\underset{K\in {𝒯}_{h}}{\sum }}h_{K}^{2}{\left|q\right|}_{1,K}^{2}\right)}^{1/2}\right); \end{array}$$

if (**v**, *q*) ∈ *V*_*h*_ × *H*_*h*_, then

(29) $$\begin{array}{c} b\left(v,q\right)\leq C\left|\left|\left|v\right|\right|\right|\cdot ||q||. \end{array}$$

**Lemma 4**

Let *Z*_*h*_ = {**v** **v** ∈ *V*_*h*_, *c*(**v**, *q*) = 0, ∀*q* ∈ *H*_*h*_}; for any **v** ∈ *Z*_*h*_, there is a constant *C*_0_ independent of *h* such that, for *α* is large enough,

(30) $$\begin{array}{c} a\left(v,v\right)\geq C_{0}{\left|\left|\left|v\right|\right|\right|}_{\mathrm{div}}^{2}. \end{array}$$

**Lemma 5**

For any *q* ∈ *H*_*h*_, there is a constant *β*_0_ independent of *h* such that

(31) $$\begin{array}{c} \underset{v\in V_{h}}{\sup}\frac{c\left(v,q\right)}{\left|\left|\left|v\right|\right|\right|}\geq \beta_{0}||q||. \end{array}$$

## 3. Existence and Uniqueness for Discontinuous Mixed Covolume Approximations

In this section, we prove that the discontinuous mixed covolume formulation has a unique solution in the finite element space *V*_*h*_ × *H*_*h*_.

**Theorem 6**

Semidiscrete discontinuous mixed covolume scheme (22) has a unique solution in the space *V*_*h*_ × *H*_*h*_.

**Proof**

Only prove that homogenous equation

(32) $$\begin{array}{c} a\left(u_{h},w\right)+b\left(w,p_{h}\right)=0,\;\forall w\in V_{h},\\ \left(p_{ht},q\right)+c\left(u_{h},q\right)=0,\;\forall q\in H_{h},\\ p_{h}\left(0\right)=0,\;u_{h}\left(0\right)=0 \end{array}$$

of (22) exists unique zero solution since the number of unknowns is the same as the number of line equations.

By letting **w** = **u**_*h*_ in the first formula of (32) and *q* = *p*_*h*_ in the second formula of (32), using Lemma 2, the sum of (32) gives

(33) $$\begin{array}{c} \left(p_{ht},p_{h}\right)+a\left(u_{h},u_{h}\right)=0. \end{array}$$

Using (1/2)(*d*/*dt*)(*p*_*h*_, *p*_*h*_) = (*p*_*ht*_, *p*_*h*_) and Lemma 4, we have

(34) $$\begin{array}{c} \frac{1}{2}\frac{d}{dt}{||p_{h}||}^{2}+C_{0}{\left|\left|\left|u_{h}\right|\right|\right|}_{\mathrm{div}}^{2}\leq 0. \end{array}$$

Integrating the above formula, we get

(35) $$\begin{array}{c} {||p_{h}||}^{2}+2C_{0}\overset{t}{\underset{0}{\int }}{\left|\left|\left|u_{h}\right|\right|\right|}_{\mathrm{div}}^{2}d\tau \leq 0. \end{array}$$

Then ||*p*_*h*_|| = 0, |||**u**_*h*_|||_div⁡_ = 0. So, *p*_*h*_ = 0, **u**_*h*_ = 0, *t* ∈ (0, *T*]. This completes the proof.

**Theorem 7**

The fully discrete discontinuous mixed covolume method defined in (24) has a unique solution in the finite element space *V*_*h*_ × *H*_*h*_.

**Proof**

Only prove that homogenous equation

(36) a(uhj,w)+b(w,phj)=0, ∀w∈Vh,(∂tphj,q)+c(uhj,q)=0, ∀q∈Hh,  (j=1,2,…,N),uh0=0, ph0=0

of (24) exists unique zero solution since the number of unknowns is the same as the number of line equations.

By letting **w** = **u**_*h*_^*j*^ in the first formula of (36) and *q* = *p*_*h*_^*j*^ in the second formula of (36), using Lemma 2, the sum of (36) gives

(37) $$\begin{array}{c} \left(\partial_{t}p_{h}^{j},p_{h}^{j}\right)+a\left(u_{h}^{j},u_{h}^{j}\right)=0,\;\left(j=1,2,\ldots ,N\right). \end{array}$$

Using Lemma 4 and

(38) (∂tphj,phj)=1Δt(phj−phj−1,phj)=12Δt[(phj,phj)−(phj−1,phj−1)   +(phj−phj−1,phj−phj−1)]=12Δt[||phj||2−||phj−1||2+||phj−phj−1||2]≥12Δt[||phj||2−||phj−1||2],

we have, from (37),

(39) ||phj||2−||phj−1||2+2C0Δt|||uhj|||div⁡2≤0, (j=1,2,…,N).

Adding the above inequality with *j* from 1 to *i*, using *p*_*h*_^0^ = 0, we have

(40) $$\begin{array}{c} {||p_{h}^{i}||}^{2}+2C_{0}\Delta t\overset{i}{\underset{j=1}{\sum }}{\left|\left|\left|u_{h}^{j}\right|\right|\right|}_{\mathrm{div}}^{2}\leq 0,\;\left(i=1,2,\ldots ,N\right). \end{array}$$

Hence, we have ||*p*_*h*_^*i*^||^2^ = 0 and |||**u**_*h*_^*j*^|||_div⁡_^2^ = 0  (*i* = 1,2,…, *N*); that is, *p*_*h*_^*i*^ = 0 and **u**_*h*_^*i*^ = 0  (*i* = 1,2,…, *N*). This completes the proof.

## 4. A Discontinuous Mixed Covolume Elliptic Projection

Define an operator *π*_*K*_ from *H* ^1^(*K*) to *P*_1_(*K*) by requiring that, for any ∀*v* ∈ *H* ^1^(*K*),

(41) $$\begin{array}{c} \int_{e_{i}}\pi_{K}v\;ds=\int_{e_{i}}v\;ds,\;\left(i=1,2,3\right), \end{array}$$

where *e*_*i*_  (*i* = 1,2, 3) are the three sides of the element *K* ∈ *𝒯*_*h*_. It was proved in [5] that

(42) $$\begin{array}{c} {\left|\pi_{K}v-v\right|}_{s,K}\leq h^{2-s}{\left|v\right|}_{2,K},\;\forall v\in H^{2}\left(K\right),\;\;\left(s=0,1,2\right). \end{array}$$

For any **v** ∈ *H*_0_^1^(*Ω*)^2^, define Π_1_ **v** ∈ *V*_*h*_ by

(43) $$\begin{array}{c} {{\left(\Pi_{1}v\right)}_{i}|}_{K}=\Pi_{K}v_{i},\;\forall K\in {𝒯}_{h},\;\;\left(i=1,2\right). \end{array}$$

Using the definition of Π_1_ and integration by parts, we can show that

(44) $$\begin{array}{c} c\left(v-\Pi_{1}v,q\right)=0,\;\forall q\in H_{h}. \end{array}$$

It was proved in [6] that

(45) $$\begin{array}{c} {\left|\left|\left|u-\Pi_{1}u\right|\right|\right|}_{\mathrm{div}}^{2}\leq ch^{2}{||u_{}^{}||}_{2}^{2}. \end{array}$$

Let Π_2_ be the projection from *L*_0_^2^(*Ω*) to the finite element space *H*_*h*_.

Define a discontinuous mixed covolume elliptic projection by requiring that, finding ${\tilde{u}}_{h},{\tilde{p}}_{n}:(0,t)\to V_{h}\times H_{h}$, such that

(46) $$\begin{array}{c} a\left(u-{\tilde{u}}_{h},w\right)+b\left(w,p-{\tilde{p}}_{h}\right)=0,\;\forall w\in V_{h},\\ c\left(u-{\tilde{u}}_{h},q\right)=0,\;\forall q\in H_{h}. \end{array}$$

It was proved in [15] that (46) has a unique solution and the error estimates in Theorem 8.

**Theorem 8**

Let $({\tilde{u}}_{h},{\tilde{p}}_{h})\in V_{h}\times H_{h}$ be the solution of (46) and let (**u**, *p*) ∈ *H* ^2^(*Ω*)^2^ × *H* ^1^(*Ω*) be the solution of (21). Then there exists a positive constant *C* independent of *h* such that

(47) $$\begin{array}{c} {\left|\left|\left|u-{\tilde{u}}_{h}\right|\right|\right|}_{\mathrm{div}}^{}+||p-{\tilde{p}}_{h}||\leq Ch\left({||u||}_{2}+{||p||}_{1}\right). \end{array}$$

**Theorem 9**

Let $({\tilde{u}}_{h},{\tilde{p}}_{h})\in V_{h}\times H_{h}$ be the solution of (46) and let (**u**, *p*) ∈ *H* ^2^(*Ω*)^2^ × *H* ^1^(*Ω*) be the solution of (21). Then there exists a positive constant *C* independent of *h* such that

(48) |||(u−u~h)t|||div⁡+||(p−p~h)t||  ≤Ch(||ut||2+||pt||1+||u||2+||p||1).

Differentiating each equation of (46) on *t* and using (44), (45), we can prove this theorem in the same way as [15].

## 5. Error Estimates for Semidiscrete Method

In this section, we will establish the error estimates in the *H*(div⁡) and *L* ^2^ norms for the semidiscrete discontinuous mixed covolume method.

**Theorem 10**

Let (**u**_*h*_, *p*_*h*_) ∈ *V*_*h*_ × *H*_*h*_ be the solution of (22) and let $p_{h}(0)={\tilde{p}}_{h}(0)$,  (**u**, *p*) ∈ *H* ^2^(*Ω*)^2^ × *H* ^1^(*Ω*) be the solution of (3). Then there exists a positive constant *C* independent of *h* such that

(49) |||u−uh|||div⁡+||p−ph|| ≤Ch[∫0t(||ut||2+||pt||1+||u||2+||p||1)dτ+||u||2+||p||1].

**Proof**

Let $\xi ={\tilde{p}}_{h}-p_{h}$, $\eta ={\tilde{u}}_{h}-u_{h}$. Subtracting the two equations of (22) from those of (21), respectively, we have

(50) $$\begin{array}{c} a\left(u-u_{h},w\right)+b\left(w,p-p_{h}\right)=0,\;\forall w\in V_{h},\\ \left(p_{t}-p_{h_{t}},q\right)+c\left(u-u_{h},q\right)=0,\;\forall q\in H_{h}. \end{array}$$

Using (46), we have

(51) $$\begin{array}{c} a\left(\eta ,w\right)+b\left(w,\xi \right)=0,\;\forall w\in V_{h},\\ \left(p_{t}-p_{h_{t}},q\right)+c\left(\eta ,q\right)=0,\;\forall q\in H_{h}. \end{array}$$

Differentiating the first equation of (51) on *t*, we have

(52) $$\begin{array}{c} a\left(\eta_{t},w\right)+b\left(w,\xi_{t}\right)=0,\;\forall w\in V_{h}. \end{array}$$

By letting *q* = *ξ*_*t*_ in the second formula of (51) and letting **w** = *η* in (52), using Lemma 2, the sum of them gives

(53) $$\begin{array}{c} a\left(\eta_{t},\eta \right)+\left(\xi_{t},\xi_{t}\right)=\left({\left({\tilde{p}}_{h}-p\right)}_{t},\xi_{t}\right). \end{array}$$

Using

(54) $$\begin{array}{c} a\left(\eta_{t},\eta \right)=\frac{1}{2}\frac{d}{dt}a\left(\eta ,\eta \right)-\frac{1}{2}\left(M_{t}\eta ,\eta \right), \end{array}$$

we have

(55) $$\begin{array}{c} \frac{1}{2}\frac{d}{dt}a\left(\eta ,\eta \right)+{||\xi_{t}||}^{2}\leq C||\xi_{t}||\cdot ||{\left({\tilde{p}}_{h}-p\right)}_{t}||+C{\left|\left|\left|\eta \right|\right|\right|}_{\mathrm{div}}^{2}. \end{array}$$

Multiplying the equation above with 2, integrating them from 0 to *t*, and using *ϵ*-inequality, Lemma 4, and (48), we can get

(56) |||η|||div⁡2+2C0(∫0t||ξt||dτ)2 ≤C1h∫0t||ξt||·(||ut||2+||pt||1+||u||2+||p||1)dτ  +C∫0t|||η|||div⁡2dt ≤1C0(∫0t||ξt||dτ)2  +Ch2(∫0t(||ut||2+||pt||1+||u||2+||p||1)dτ)2  +C∫0t|||η|||div⁡2dt;

then

(57) |||η|||div⁡2+1C0(∫0t||ξt||dτ)2  ≤Ch2(∫0t(||ut||2+||pt||1+||u||2+||p||1)dτ)2,

so

(58) $$\begin{array}{c} {\left|\left|\left|\eta \right|\right|\right|}_{\mathrm{div}}^{}\leq Ch^{}\overset{t}{\underset{0}{\int }}\left({||u_{t}||}_{2}+{||p_{t}||}_{1}+{||u||}_{2}+{||p||}_{1}\right)d\tau , \end{array}$$

(59) $$\begin{array}{c} \overset{t}{\underset{0}{\int }}||\xi_{t}||d\tau \leq Ch\overset{t}{\underset{0}{\int }}\left({||u_{t}||}_{2}+{||p_{t}||}_{1}+{||u||}_{2}+{||p||}_{1}\right)d\tau ; \end{array}$$

hence

(60) ||ξ||≤∫0t||ξt||dτ≤Ch∫0t(||ut||2+||pt||1+||u||2+||p||1)dτ.

Now, using the triangle inequalities (47), (58), and (60), we get

(61) |||u−uh|||div⁡+||p−ph|| ≤Ch[∫0t(||ut||2+||pt||1+||u||2+||p||1)dτ+||u||2+||p||1].

The proof is complete.

## 6. Error Estimates for Fully Discrete Method

Let $\xi^{j}={\tilde{p}}_{h}^{j}-p_{h}^{j}$, $\zeta^{j}={\tilde{p}}_{h}^{j}-p^{j}$, $\eta^{j}={\tilde{u}}_{h}^{j}-u_{h}^{j}\;\;\left(j=0,1,\ldots ,N\right)$; then the error estimates for the backward Euler fully discrete discontinuous mixed covolume method in the *H*(div⁡) and *L* ^2^ norms are provided in the next two theorems.

**Theorem**

Let (**u**, *p*) ∈ *H* ^2^(*Ω*)^2^ × *H* ^1^(*Ω*) be the solution of (3), and let (**u**_*h*_^*j*^, *p*_*h*_^*j*^) ∈ *V*_*h*_ × *H*_*h*_  (*j* = 1,2,…, *N*) be the solution of (24) with *t* = *t* ^*j*^  (*j* = 1,2,…, *N*), respectively. If $p_{h}^{0}={\tilde{p}}_{h}(0)=p_{0}$, $u_{h}^{0}={\tilde{u}}_{h}(0)=u_{0}$, then there exists a positive constant C independent of *h* and Δ*t* such that

(62) max⁡0≤i≤N||pi−phi|| ≤CΔt||ptt||L∞(L2)  +Ch(||ut||L∞(H2)+||pt||L∞(H1)+||u||L∞(H2)     + ||p||L∞(H1)).

**Proof**

Subtracting the two equations of (24) from (21), respectively, with *t* = *t* ^*j*^  (*j* = 0,1,…, *N*), we can get the error equation:

(63) a(uj−uhj,w)+b(w,pj−phj)=0, ∀w∈Vh,(ptj−∂tphj,q)+c(uj−uhj,q)=0,∀q∈Hh, (j=1,2,…,N).

Choosing **w** = *η* ^*j*^  (*j* = 1,2,…, *N*) and *q* = *ξ* ^*j*^  (*j* = 1,2,…, *N*) in the two equations of (63), adding them together, and using Lemma 2, discontinuous mixed covolume elliptic projection, we have

(64) $$\begin{array}{c} a\left(\eta^{j},\eta^{j}\right)+\left(\partial_{t}\xi^{j},\xi^{j}\right)=\left(\partial_{t}p^{j}-p_{t}^{j},\xi^{j}\right)+\left(\partial_{t}\zeta^{j},\xi^{j}\right). \end{array}$$

First, we estimate the left item of (64). Using Lemma 4, we have

(65) a(ηj,ηj)≥C0|||ηj|||div⁡2,(∂tξj,ξj) =1Δt(ξj−ξj−1,ξj) =12Δt[(ξj,ξj)−(ξj−1,ξj−1)+(ξj−ξj−1,ξj−ξj−1)] =12Δt[||ξj||2−||ξj−1||2+||ξj−ξj−1||2] >12Δt[||ξj||2−||ξj−1||2].

Then, we estimate the right item of (64). From

(66) ||∂tpj−ptj||2 =||1Δt∫tj−1tj(tj−1−t)pttdt||2 ≤∫Ω(1Δt∫tj−1tj(tj−1−t)pttdt)2dχ ≤1(Δt)2∫Ω(∫tj−1tj(tj−1−t)2dt∫tj−1tjptt2dt)dχ ≤C(Δt)2||ptt||L∞(L2)2,

we have

(67) (∂tpj−ptj,ξj)≤C(Δt)2||ptt||L∞(L2)2+C||ξj||2,||∂tζj||=||ζj−ζj−1Δt||=||1Δt∫tj−1tjζtdt||≤1Δt∫tj−1tj||ζt||dt≤ChΔt∫tj−1tj(||ut||2+||pt||1+||u||2+||p||1)dt≤Ch(||ut||L∞(H2)+||pt||L∞(H1)+||u||L∞(H2)   + ||p||L∞(H1));

therefore

(68) (∂tζj,ξj) ≤Ch2(||ut||L∞(H2)2+||pt||L∞(H1)2+||u||L∞(H2)2     + ||p||L∞(H1)2)2+C||ξj||2.

Substituting the estimations above into (64), using *ξ* ^0^ = 0, we have

(69) ||ξi||2+2C0Δt∑j=1i|||ηj|||div⁡2 ≤C(Δt)2||ptt||L∞(L2)2+CΔt∑j=1i||ξj||2  +Ch2(||ut||L∞(H2)+||pt||L∞(H1)+||u||L∞(H2)+||p||L∞(H1))2.

By *ϵ*-inequality and the discrete Gronwall inequality, we have

(70) max⁡0≤i≤N||ξi|| ≤CΔt||ptt||L∞(L2)  +Ch(||ut||L∞(H2)+||pt||L∞(H1)+||u||L∞(H2)+||p||L∞(H1)).

From the above formula and (47) and using the triangle inequality, we have

(71) max⁡0≤i≤N||pi−phi|| ≤CΔt||ptt||L∞(L2)  +Ch(||ut||L∞(H2)+||pt||L∞(H1)+||u||L∞(H2)+||p||L∞(H1)).

This completes the proof.

**Theorem 12**

Let (**u**, *p*) ∈ *H* ^2^(*Ω*)^2^ × *H* ^1^(*Ω*) be the solution of (3), and let (**u**_*h*_^*j*^, *p*_*h*_^*j*^) ∈ *V*_*h*_ × *H*_*h*_  (*j* = 1,2,…, *N*) be the solution of (24) with *t* = *t* ^*j*^  (*j* = 1,2,…, *N*), respectively. If $p_{h}^{0}={\tilde{p}}_{h}(0)=p_{0}$, $u_{h}^{0}={\tilde{u}}_{h}(0)=u_{0}$, then there exists a positive constant C independent of *h* and Δ*t* such that

(72) max⁡0≤i≤N|||ui−uhi|||div⁡ ≤CΔt||ptt||L∞(L2)  +Ch(||ut||L∞(H2)+||pt||L∞(H1)+||u||L∞(H2)+||p||L∞(H1)).

**Proof**

Choosing **w** = *η* ^*j*^  (*j* = 1,2,…, *N*) and *q* = ∂_*t*_ *ξ* ^*j*^  (*j* = 1,2,…, *N*) in the two equations of (63), adding them together, and using Lemma 2, discontinuous mixed covolume elliptic projection, we have

(73) $$\begin{array}{c} a\left(\partial_{t}\eta^{j},\eta^{j}\right)+\left(\partial_{t}\xi^{j},\partial_{t}\xi^{j}\right)=\left(\partial_{t}p^{j}-p_{t}^{j},\partial_{t}\xi^{j}\right)+\left(\partial_{t}\zeta^{j},\partial_{t}\xi^{j}\right). \end{array}$$

From

(74) $$\begin{array}{c} a\left(\partial_{t}\eta^{j},\eta^{j}\right)=\frac{1}{2}\partial_{t}a\left(\eta^{j},\eta^{j}\right)+\frac{\Delta t}{2}a\left(\partial_{t}\eta^{j},\partial_{t}\eta^{j}\right)\geq C_{0}{\left|\left|\left|\eta^{j}\right|\right|\right|}_{\mathrm{div}}^{2} \end{array}$$

and (66) and (67), we have

(75) |||ηj|||div⁡2 ≤C(Δt)2||ptt||L∞(L2)  +Ch2(||ut||L∞(H2)+||pt||L∞(H1)+||u||L∞(H2)+||p||L∞(H1))2;

therefore

(76) |||ηj|||div⁡ ≤CΔt||ptt||L∞(L2)  +Ch(||ut||L∞(H2)+||pt||L∞(H1)+||u||L∞(H2)+||p||L∞(H1))2.

From the above formula and (47) and using the triangle inequality, we have

(77) max⁡0≤i≤N|||ui−uhi|||div⁡ ≤CΔt||ptt||L∞(L2)  +Ch(||ut||L∞(H2)+||pt||L∞(H1)+||u||L∞(H2)+||p||L∞(H1)).

The proof is complete.
